# Yunnan Baiyao Adjuvant Treatment for Patients with Hemoptysis: A Systematic Review and Meta-Analysis

**DOI:** 10.1155/2022/4931284

**Published:** 2022-02-22

**Authors:** Yilan Wang, Xiaomin Wang, Hongfang Fu, Shuo Kou, Demei Huang, Zherui Shen, Fei Wang, Zhenxing Wang

**Affiliations:** ^1^Hospital of Chengdu University of Traditional Chinese Medicine, Chengdu 610072, Sichuan Province, China; ^2^Zhejiang University of Traditional Chinese Medicine, Hangzhou 310053, Zhejiang Province, China

## Abstract

**Background:**

Yunnan Baiyao (YNBY) is a traditional Chinese medicine used to treat bleeding. We evaluated the efficacy of YNBY plus conventional pharmaceutical treatment (CPT) versus CPT alone in patients with hemoptysis.

**Methods:**

A total of eight electronic databases were searched. The outcomes in the included studies were effective rate, hemoptysis volume, duration of hemoptysis and hospitalization, number of cases requiring endotracheal intubation, and adverse events (AEs). The studies were used to calculate risk ratios (RRs) or mean differences (MDs) with corresponding 95% confidence intervals. Risk of bias for included trials was assessed using the Cochrane risk of bias tool.

**Results:**

Thirteen RCTs were analyzed consisting of a total of 1379 patients. Treatment with YNBY + CPT had a greater effective rate than CPT alone (RR: 1.18; 95% CI: 1.13 to 1.23; *P* < 0.001; *I*^2^ = 0%), a lower hemoptysis volume (MD: −107.37; 95% CI: −121.69 to −93.06; *P* < 0.001; *I*^2^ = 0%), a shorter duration of hemoptysis (MD: −2.70; 95% CI: −2.96 to 2.43; *P* < 0.001; *I*^2^ = 0%) and hospitalization (MD: −2.38; 95% CI: −2.93 to −1.83; *P* < 0.001; *I*^2^ = 9%), and a reduction in the incidence of AEs (RR: 0.34; 95% CI: 0.23 to 0.51; *P* < 0.001; *I*^2^ = 0%). YNBY + CPT treatment provided no significant difference in reducing the number of cases requiring endotracheal intubation compared to CPT alone (RR: 0.49; 95% CI: 0.15 to 1.60; *P*=0.24; *I*^2^ = 0%).

**Conclusion:**

YNBY plus CPT showed better efficacy than CPT for patients with hemoptysis. Our study provides medical evidence for the efficacy and safety of YNBY for hemoptysis.

## 1. Introduction

Hemoptysis is a common symptom of lung, bronchial, and circulatory diseases [1, 2]. The recurrence rate of hemoptysis is 22% [3, 4], and the mortality rate of patients with massive hemoptysis is approximately 15% [5]. If hemoptysis-induced asphyxia does not receive prompt treatment, it may cause death [3]. In Europe, bronchiectasis, malignancy, pneumonia, posttuberculosis sequelae, and idiopathic hemorrhage are considered to be the most common causes of hemoptysis [6–10]. In the United States, 7 to 16% of tuberculosis patients experience hemoptysis [11]. In China, the most common causes of hemoptysis are tuberculosis, bronchiectasis, bronchial cancer, and lung abscesses [12]. More than 70% of Chinese patients with bronchiectasis experience hemoptysis [13]. The extent of hemoptysis can also reflect the severity of the bronchiectasis, affecting the patient's prognosis [13].

Treatment for hemoptysis includes normal saline lavage, local infusion of vasoconstrictors, combined therapy of fibrinogen and thrombin under endoscopy, bronchial artery embolization, and drug treatments [3, 14, 15]. Conventional treatment using hemoptysis drugs can result in adverse reactions (AEs) [16–19].

Traditional Chinese medicine (TCM) evolved as a system of medical practice from ancient China more than 2000 years ago. It is a holistic system of medicine that integrates prevention, treatment, and rehabilitation. Much effort has been made to describe the complicated system of TCM, with a combination of its traditional treatments and modern technological means [20]. In China, a plenty of research studies showed that integrative medicine therapy has shown more advantages in many diseases than using Western medicine alone, in the aspects of increasing efficacy and reducing side effects [21, 22]. Meanwhile, modern chemical, randomized clinical trials and molecular biological experiments were carried out with the traditional medicine for further pathological mechanism of diseases in the world [23–25]. Yunnan Baiyao (YNBY) is a Chinese herbal medicinal formula developed in 1902. It is used widely for its hemostatic effect and has been approved by the China Food and Drug Administration [26] (CFDA) (approval no. Z53020799). YNBY formulations come in many forms, including powder, capsules, aerosol, tincture, plaster, hemorrhoid cream, and toothpaste [26]. The known medicinal ingredients of YNBY have been reported [27, 28]. Studies have shown that components of YNBY enhance hemostasis, reduce stress-related inflammation, and increase antioxidant activity [27, 29–32]. *Panax notoginseng* is the main herb in YNBY, and its active compounds have therapeutic effects on numerous conditions, including cerebral ischemia-reperfusion, atherosclerosis, renal fibrosis, tumors, hypertension, myocardial ischemia, Alzheimer's disease, and skin wound bleeding [33–35]. *Panax notoginseng* significantly increases the number of platelets in peripheral blood, enhances platelet adhesion, and establishes hemostasis [29, 36–38]. Other drugs of YNBY include *Radix Aconiti Kusnezoffii*, *Borneolum Synthcticum*, *Forest Musk*, and *Rhizoma Paridis* (*Chonglou*). In addition, the complete formula composition of YNBY is kept secret and not available to the public [39].

Randomized clinical trials (RCTs) have demonstrated that YNBY is effective in the treatment of hemoptysis in patients [40–43]. The purpose of this systematic review and meta-analysis is to assess the effectiveness and safety of YNBY across RCTs investigating hemoptysis treatments and to provide a summary of evidence and guidance for the clinical use and continued research of YNBY. The purpose of the study was expressed in a form of PICO framework, which is uploaded as Supplementary File 1.

## 2. Methods

### 2.1. Search Strategy

This study has been registered on INPLASY (https://inplasy.com/inplasy-2021-7-0007/, registration no. INPLASY202170007). This review was conducted according to [44] Preferred Reported Items for Systematic Reviews and Meta-Analysis (PRISMA) guidelines. The Cochrane Library, Web of Science, Embase, PubMed, China National Knowledge Infrastructure (CNKI), Chongqing VIP Chinese Science and Technology Periodical Database, the Sinomed database, and the Wanfang database were searched from inception to June 30, 2021, for RCTs investigating the clinical efficacy of YNBY. Two of us extracted data from the RCTs. Conflicts were resolved through discussion with a third author. The following keywords were used in the search: (1) Hemoptysis OR hemoptyses OR hemoptoe OR spit blood OR hemoptysis OR coughing up blood OR emptysis; (2) Yunnanbaiyao OR Yunnan Baiyao OR Yun nan bai yao OR ynby OR Baiyao OR Yunnanbaiyao capsule OR Yunnanbaiyao capsules; (3) trial OR clinical trials OR clinical trial OR random OR random allocation OR therapeutic use OR randomized controlled trial OR RCT; (4) #1 AND #2 AND #3. Various combinations of the terms were used for different databases, as shown in Supplementary File 2. In addition, we searched online trial registries including the Chinese Clinical Trial Registry and ClinicalTrials.gov trials to avoid missing any relevant RCTs.

### 2.2. Selection Criteria

The inclusion criteria were as follows: (1) patients diagnosed with hemoptysis; (2) patients in both test and control groups were given cough relief, expectorant, bed rest, anti-infection, phentolamine or pituitrin, and other conventional treatments; (3) the YNBY group was treated with only YNBY + conventional pharmaceutical therapy (CPT), and the control group was treated with only the CPT; (4) YNBY was given orally; and (5) one or more of the following outcomes were investigated: effective rate, hemoptysis volume, duration of hemoptysis, duration of hospitalization, number of cases requiring endotracheal intubation, and AEs. Considering the illness duration of hemoptysis was different according to the etiologies, and the treatment duration depended on hemoptysis manifestations and the duration of hemoptysis; the clinical trials consisting of all different treatment durations were included.

The exclusion criteria were as follows: (1) diseases that did not meet the diagnostic criteria for hemoptysis; (2) the use of surgery or treatments with other traditional Chinese medicines and/or CPTs; (3) reviews, conference abstracts, and studies that included animal experiments; (4) duplicate publications; and (5) incomplete or incorrect data.

### 2.3. Data Extraction and Assessment of the Risk of Bias

Two reviewers independently extracted and cross-checked RCT data according to the inclusion criteria. Disagreements were resolved by a third researcher. The data extracted included the subject, the first author, publication time, basic characteristics of the subjects, sample size, the intervention measures of the experimental and control groups, drug dose, course of treatment, and outcome indices.

Risk of bias for included trials was assessed using the Cochrane risk of bias tool. The two independent reviewers evaluated random sequence generation, allocation concealment, blinding of participants and personnel, blinding of outcome assessments, incomplete outcome data, selective reporting, and other biases in each RCT. Each item was classified as low risk, high risk, or unclear risk [45]. Any disagreements were resolved by a discussion among all reviewers.

### 2.4. Statistical Analysis

Statistical analyses were performed using RevMan 5.3 and Stata 14.0. Risk ratios (RRs) and 95% confidence intervals were used for dichotomous data. Mean difference (MD) and 95% CIs were used for continuous data. The heterogeneity among the studies was assessed using the *χ*^2^ test and *I*^2^ statistics [46]. If *P* > 0.10 and *I*^2^  <  50%, the study was considered to have no statistical heterogeneity, and a fixed-effect model was used in the analysis. If *P* < 0.10 and *I*^2^  >  50%, a study was considered to be statistically heterogeneous, and a random-effect model was used in the analysis. The results of the meta-analysis were presented as forest maps. Subgroup analysis was used to determine any source of heterogeneity. Due to different efficacy evaluation criteria in the RCTs, we conducted subgroup analyses based on predefined variables. If there were 10 or more studies in any group or subgroup, Begger's test was performed, and funnel plots were generated to assess publication bias. Sensitivity analysis (SA) was conducted by excluding RCTs one by one and comparing analysis results with those before the RCTs' exclusions.

## 3. Results

### 3.1. Study Selection

**Figure 1:**
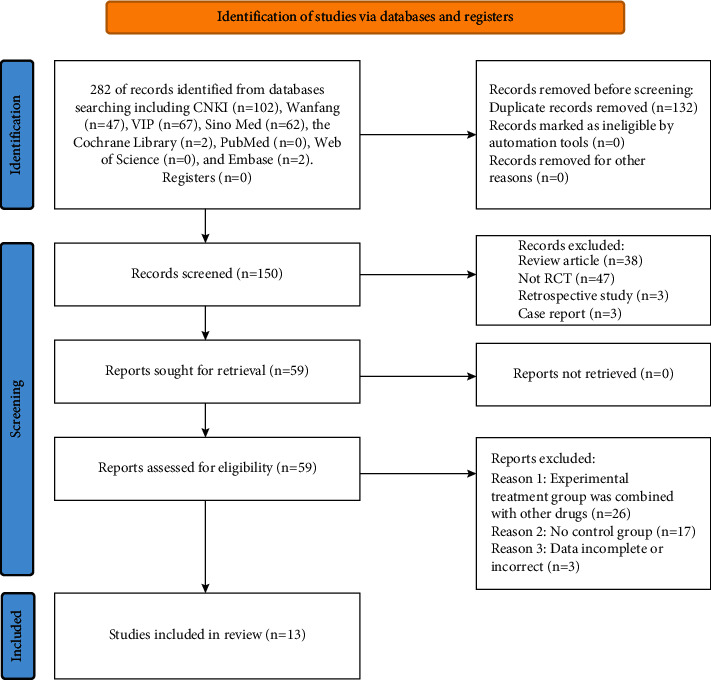
PRISMA flow diagram of the study.

One hundred and two studies were retrieved from CNKI, 47 studies were retrieved from Wanfang Journal Database, 67 studies were retrieved from VIP Database, 62 studies were retrieved from Sinomed, 2 studies were retrieved from the Cochrane Library, 0 studies were retrieved from PubMed, 0 studies were retrieved from the Web of Science, and 2 studies were retrieved from Embase for a total of 282 studies (Figure 1). After deleting duplicates, 150 studies remained. There were 91 unqualified articles excluded for not meeting the inclusion criteria after title and abstract review. Additional 46 studies were excluded after reading the full text (Figure 1).

### 3.2. Study Characteristics

Table 1 lists the characteristics of the patients in the RCTs [22, 40, 41, 47–56]. All RCTs compared Yunnan Baiyao with and without CPT. The treatment duration varies from 3 days to 2 weeks in our included study. In 6 RCTs [41, 47, 51–53, 56], the standard deviations in the ages of the experimental and the control groups were not reported, but they did report that the difference in mean ages between the experimental and control groups was not statistically significant.

### 3.3. Methodological Quality

**Figure 2:**
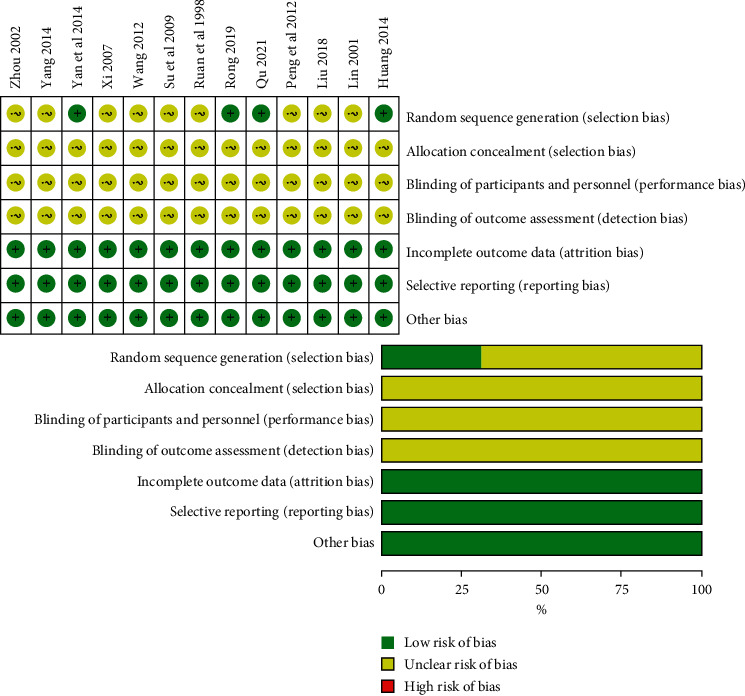
Risk of bias assessment of the trials included in the study. “?” = unclear risk; “+” = low risk.

Of the 13 RCTs [22, 40, 41, 47–56], 4 [40, 41, 47, 50] used the random number table method for randomization. None of the trials reported allocation concealment, blinding of participants and personnel, or blinding of outcomes' assessment. There was no selective outcome reporting or case shielding in the 13 RCTs. No other biases were reported. The risk of bias assessment data are shown in Figure 2.

### 3.4. Primary Outcome

#### 3.4.1. Effective Rate

**Figure 3:**
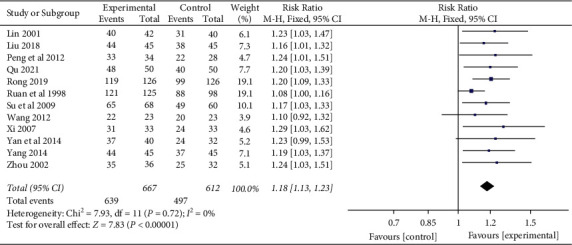
Risk ratio and 95% confidence interval (CI) of the meta-analysis of the effect of Yunnan Baiyao on the effective rate of remission of hemoptysis symptoms related to twelve randomized clinical trials included in the study. Events = the number of all symptom remission cases; total = total number of cases; Huang [47] study was not included in the meta-analysis of effective rate because the effective rate was not reported in the study.

**Figure 4:**
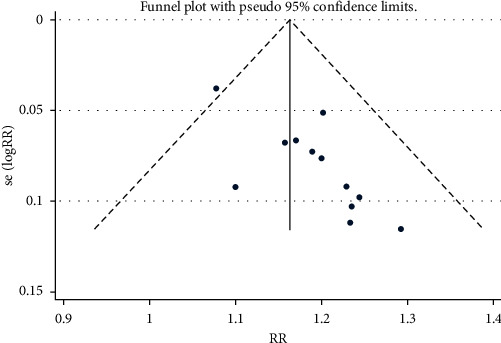
Funnel plot for the publication bias of effective rate. RR: risk ratio.

Twelve [22, 40, 41, 48–56] of the 13 studies evaluated the effective rate of YNBY + CPT treatment compared to CPT alone (Figure 3). There was no heterogeneity among the 12 studies (*χ*^2^ = 7.93; *P*=0.72; *I*^2^ = 0%). A fixed-effect model was used for the analyses when no heterogeneity was detected among a set of studies. The effective rate of YNBY + CPT treatment was significantly higher than that of CPT treatment alone (RR: 1.18; 95% CI: 1.13 to 1.23; *P* < 0.001, Figure 3). Begger's test and resulting funnel plots revealed that the distribution of scattered points on both sides of the equivalence line is symmetric, and the points primarily distributed in the middle and at the top of the graph (Figure 4). It is known that *P* < 0.05 indicated publication bias, while >0.05 indicated no publication bias [57]. The results indicated that there was no publication bias in the included studies (*P*=0.64, Figure 4).

**Figure 5:**
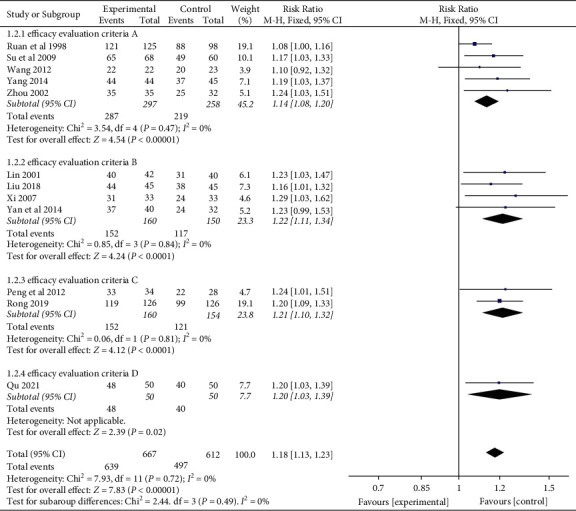
Risk ratio and 95% confidence interval (CI) of the subgroup meta-analysis of the effect of Yunnan Baiyao on the effective rate of remission of hemoptysis symptoms based on different efficacy evaluation criteria. Events = the number of all symptom remission cases; total = total number of cases.

Different subgroup analyses were conducted according to different efficacy evaluation criteria in these RCTs. It should be noted that, in these RCTs, the time range of efficacy evaluation criteria is independent of the time range of treatment duration. The efficacy evaluation criteria A of 5 studies [51–55] were as follows: clinical recovery: hemoptysis does not last more than 1 week with treatment; markedly effective: hemoptysis is controlled within 1 week with treatment, with occasional blood in sputum; effective: hemoptysis is significantly reduced within 1 week with treatment; and invalid: hemoptysis is not significantly reduced in 1 week with treatment. There was no heterogeneity among the included studies (*χ*^2^ = 3.54; *P*=0.47; *I*^2^ = 0%, Figure 5). The effective rate of YNBY + CPT treatment was significantly higher than that of CPT treatment alone (RR: 1.14; 95% CI: 1.08 to 1.20; *P* < 0.001, Figure 5).

The efficacy evaluation criteria B of 4 studies [22, 41, 48, 54] were as follows: markedly effective: hemoptysis does not last more than 24 hours with treatment; effective: the duration of hemoptysis does not exceed 48 hours with treatment; and invalid: patients with hemoptysis persisted for more than 48 hours with treatment. There was no heterogeneity among the included studies (*χ*^2^ = 0.85; *P*=0.84; *I*^2^ = 0%, Figure 5). The effective rate of YNBY + CPT treatment was significantly higher than that of CPT treatment alone (RR: 1.22; 95% CI: 1.11 to 1.34; *P* < 0.001, Figure 5).

The efficacy evaluation criteria C of 2 studies [49, 50] were as follows: markedly effective: hemoptysis stops within 5 days with treatment; effective: the interval between hemoptysis is prolonged, or there is no massive hemoptysis, with occasional blood in sputum within 5 days with treatment; and invalid: patients with hemoptysis persisted for more than 5 days with treatment. There was no heterogeneity among the included studies (*χ*^2^ = 0.06; *P*=0.81; *I*^2^ = 0%, Figure 5). The effective rate of YNBY + CPT treatment was significantly higher than that of CPT treatment alone (RR: 1.21; 95% CI: 1.10 to 1.32; *P* < 0.001, Figure 5).

The efficacy evaluation criteria D of 1 study [40] were as follows: markedly effective: hemoptysis stops within 3 days with treatment; effective: hemoptysis is reduced by more than 50% within 3 days with treatment; and invalid: patients with hemoptysis show no improvement within 3 days with treatment. The effective rate of YNBY + CPT treatment was significantly higher than that of CPT treatment alone (RR: 1.20; 95% CI: 1.03 to 1.39, *P*=0.02, Figure 5).

**Figure 6:**
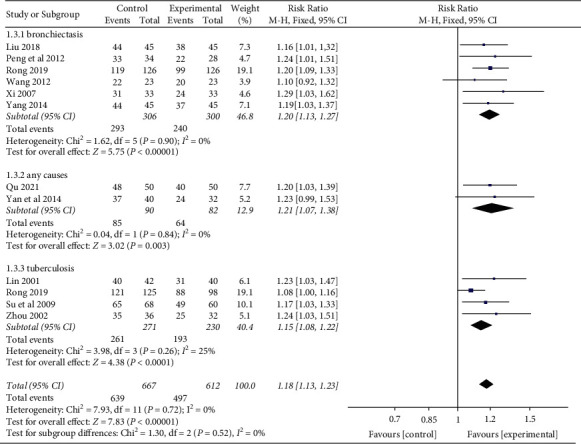
Risk ratio and 95% confidence interval (CI) of the subgroup meta-analysis of the effect of Yunnan Baiyao on the effective rate of remission of hemoptysis symptoms based on different causes of hemoptysis. Events = the number of all symptom remission cases; total = total number of cases.

In addition, we performed a subgroup analysis based on different causes of hemoptysis, which included bronchiectasis, tuberculosis, and any causes. The results showed that no significant differences were found in different causes of hemoptysis (*P*=0.52) subgroups, as shown in Figure 6.

### 3.5. Secondary Outcomes

#### 3.5.1. Hemoptysis Volume (cc)

**Figure 7:**
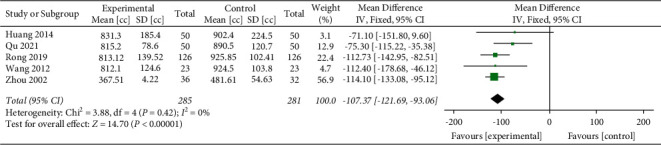
Mean differences and 95% confidence intervals (CIs) of the meta-analysis of the effect of Yunnan Baiyao on the hemoptysis volume (cc) related to the five randomized clinical trials included in the study. Total = total number of cases; Lin [48], Liu [22], Peng [49], Ruan [51], Su [52], Xi [54], Yan [41], and Yang [55] studies were not included in the meta-analysis of hemoptysis volume because the hemoptysis volume was not reported in the studies.

Five studies [40, 47, 50, 53, 56] evaluated the efficacy of YNBY + CPT treatment on hemoptysis volume compared to CPT alone. There was no heterogeneity among the included studies (*χ*^2^ = 3.88; *P*=0.42; *I*^2^ = 0%). A fixed-effect model was used for the analyses when no heterogeneity was detected among a set of studies. The hemoptysis volume of the YNBY + CPT treatment was significantly less than that of CPT treatment alone (MD: −107.37 cc; 95% CI: −121.69 to −93.06 cc; *P* < 0.001, Figure 7).

**Figure 8:**
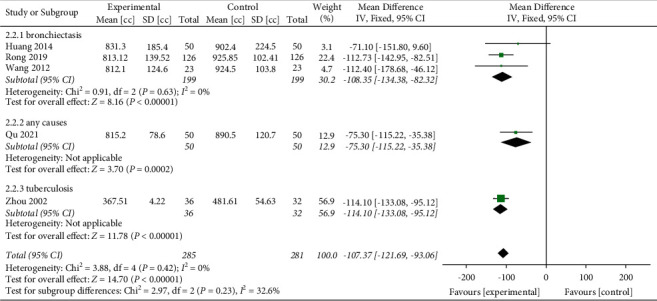
Mean differences and 95% confidence intervals (CIs) of the subgroup meta-analysis of the effect of Yunnan Baiyao on the hemoptysis volume (cc) based on different causes of hemoptysis. Total = total number of cases.

Considering that hemoptysis may be caused by different types of diseases, we performed a subgroup analysis. The results indicated that no significant differences were found in different causes of hemoptysis (*P*=0.23) subgroups, as shown in Figure 8.

#### 3.5.2. Duration of Hemoptysis (Day)

**Figure 9:**
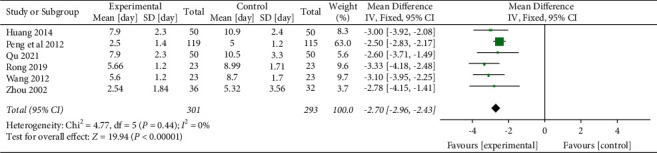
Mean differences and 95% confidence intervals (CIs) of the meta-analysis of the effect of Yunnan Baiyao on the duration of hemoptysis (day) related to the six randomized clinical trials included in the study. Total = total number of cases; Lin [48], Liu [22], Ruan [51], Su [52], Xi [54], Yan [41], and Yang [55] studies were not included in the meta-analysis of the duration of hemoptysis because the duration of hemoptysis was not reported in the studies.

Six studies [40, 47, 49, 50, 53, 56] evaluated the efficacy of YNBY + CPT treatment on the duration of hemoptysis compared to CPT alone. There was no heterogeneity among the included studies (*χ*^2^ = 4.77; *P*=0.44; *I*^2^ = 0%). The duration of hemoptysis of the YNBY + CPT treatment was significantly shorter than that of CPT treatment alone (MD: −2.70 days; 95% CI: −2.96 to 2.43 days; *P* < 0.001, Figure 9).

**Figure 10:**
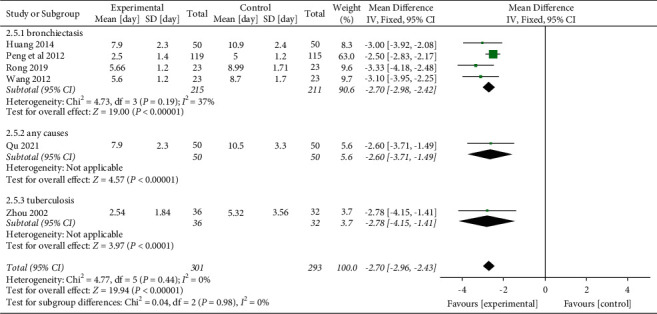
Mean differences and 95% confidence intervals (CIs) of the subgroup meta-analysis of the effect of Yunnan Baiyao on the duration of hemoptysis (day) based on different causes of hemoptysis. Total = total number of cases.

Meanwhile, we performed a subgroup analysis based on the causes of hemoptysis. The results demonstrate that no significant differences were found in different causes of hemoptysis (*P*=0.98) subgroups, as shown in Figure 10.

#### 3.5.3. Duration of Hospitalization (day)

**Figure 11:**
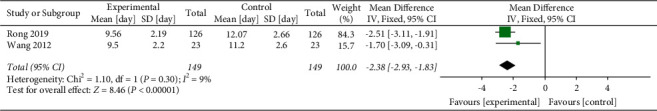
Mean differences and 95% confidence intervals (CIs) of the meta-analysis of the effect of Yunnan Baiyao on the duration of hospitalization (day) of the patients with hemoptysis related to the meta-analysis of two randomized clinical trials included in the study. Total = total number of cases. Huang [47], Lin [48], Liu [22], Peng [49], Qu [40], Ruan [51], Su [52], Xi [54], Yan [41], Yang [55], and Zhou [56] studies were not included in the meta-analysis of the duration of hospitalization because the duration of hospitalization was not reported in the studies.

Two studies [50, 53] evaluated the efficacy of YNBY + CPT treatment on the duration of hospitalization compared to CPT alone. There was no heterogeneity between the included studies (*χ*^2^ = 1.10; *P*=0.30; *I*^2^ = 9%). The duration of hospitalization of the YNBY + CPT treatment was significantly shorter than that of CPT treatment alone (MD: −2.38 days; 95% CI: −2.93 to −1.83 days; *P* < 0.001, Figure 11).

#### 3.5.4. Number of Cases Requiring Endotracheal Intubation

**Figure 12:**
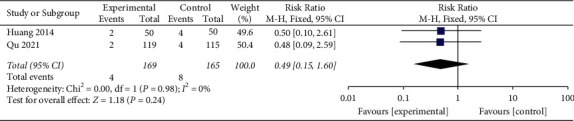
Risk ratio and 95% confidence interval (CI) of the meta-analysis of the effect of Yunnan Baiyao on the number of cases requiring endotracheal intubation among the patients with hemoptysis related to the two randomized clinical trials included in the study. Events = the number of cases requiring endotracheal intubation; Total = total number of cases; Lin [48], Liu [22], Peng [49], Rong [50], Ruan [51], Su [52], Wang [53], Xi [54], Yan et al., 2014, Yang [55], and Zhou [56] studies were not included in the meta-analysis of the number of cases requiring endotracheal intubation because the number of cases requiring endotracheal intubation was not reported in the studies.

Two studies [40, 47] evaluated the efficacy of YNBY + CPT treatment on the number of cases requiring endotracheal intubation compared to CPT alone. There was no heterogeneity between the included studies (*χ*^2^ = 0.00; *P*=0.98; *I*^2^ = 0%). There was no statistical difference in the number of patients requiring endotracheal intubation in the YNBY + CPT treatment compared to CPT treatment alone (*Z* = 1.18; *P*=0.24, Figure 12).

#### 3.5.5. Adverse Events

**Figure 13:**
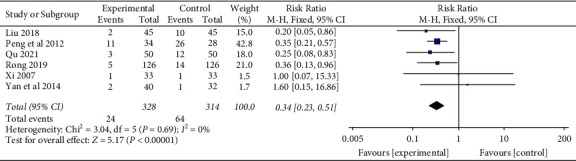
Risk ratio and 95% confidence interval (CI) of the meta-analysis of the effect of Yunnan Baiyao on the adverse events of the patients with hemoptysis related to the meta-analysis of six randomized clinical trials included in the study. Events = the number of cases of adverse events; Total = total number of cases; Huang [47], Lin [48], Ruan [51], Su [52], Wang [53], Yang [55], and Zhou [56] studies were not included in the meta-analysis of adverse events because the adverse events were not reported in the studies.

Six studies [22, 40, 41, 49, 50, 54] evaluated adverse events (AEs) of YNBY + CPT treatment compared to CPT alone. The 6 studies reported AEs, including nausea, abdominal pain, chest tightness, dizziness, hyponatremia, hypertension, and rash. There was no heterogeneity among the 6 studies (*I*^2^ = 0%; *P*=0.69). The incidence of AEs in the YNBY + CPT treatment was significantly lower than that of CPT treatment alone (RR: 0.34; 95% CI: 0.23 to 0.51; *P* < 0.001, Figure 13).

**Figure 14:**
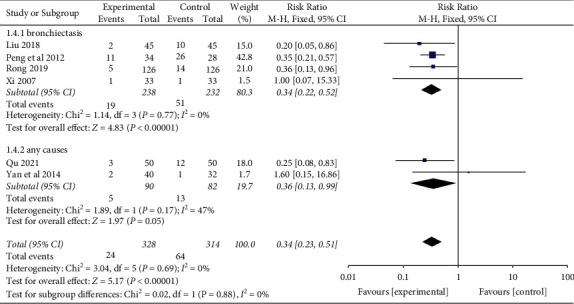
Risk ratio and 95% confidence interval (CI) of the subgroup meta-analysis of the effect of Yunnan Baiyao on the adverse events of the patients with hemoptysis based on different causes of hemoptysis. Events = the number of cases of adverse events; total = total number of cases.

We conducted a subgroup analysis according to the causes of hemoptysis. The results demonstrate that no significant differences were found in different causes of hemoptysis (*P*=0.88) subgroups, as shown in Figure 14.

## 4. Sensitivity Analysis

SA was performed to assess the robustness of the pooled results by excluding all included studies one by one. There were no significant differences between the original meta-analysis and the SA in terms of the effective rate, hemoptysis volume, duration of hemoptysis, duration of hospitalization, number of cases requiring endotracheal intubation, and incidence of adverse events. The sensitivity of the analysis to differences in effective rates, hemoptysis volumes, durations of hemoptysis, durations of hospitalization, numbers of cases requiring endotracheal intubation, and incidences of adverse events was low, and the results were stable.

## 5. Discussion

The 13 RCTs in this meta-analysis included a total of 1379 patients that were distributed among 13 provinces and cities in China, from different ethnic groups, age groups, and genders. Confounding factors such as smoking, malignancy, pulmonary infection, and anticoagulant drug use were not fully reported in the RCTs analyzed in this study. There was no significant heterogeneity among RCTs of differing efficacy evaluation criteria nor subgroups of RCTs with similar efficacy evaluation criteria.

In this study, we assessed the efficacy and safety of YNBY + CPT treatment of patients with hemoptysis. YNBY + CPT treatment resulted in a higher effective rate, a lower hemoptysis volume, a shorter duration of hemoptysis, and a shorter duration of hospitalization compared to CPT treatment alone. Yunnan Baiyao may reduce the mortality of hemoptysis-induced asphyxiation by reducing the amount of hemoptysis. The duration of hospitalization is correlated to the cost of hospitalization because the increased duration of hospitalization imposes an economic burden to patients. Yunnan Baiyao can shorten the duration of hospitalization and reduce the economic burden caused by prolonged hospitalization, so it provides economic value. YNBY + CPT treatment significantly reduced the incidence of AEs compared to CPT treatment alone. However, YNBY + CPT treatment provided no significant difference in reducing the number of cases requiring endotracheal intubation compared to CPT alone. Hemoptysis patients eventually requiring endotracheal intubation are generally sicker and appear to be recalcitrant to YNBY treatment. Yunnan Baiyao may not be suitable for patients suffering massive hemoptysis, but it may be successfully administered to patients with mild to moderate hemoptysis. Our intubation results may be due to the small population sizes assessed in the RCTs. Larger clinical trials may provide more definite results.

Hemoptysis is one of the most common complications of respiratory and circulatory system diseases [2] with high mortality and recurrence rates [58], but there are no clear guidelines for the treatment of hemoptysis. Vasopressin is commonly used in clinical studies of hemoptysis treatments [59–62]. Because of its strong vasoconstriction effects, vasopressin is effective for controlling recurrent hemoptysis, but vasopressin use results in many AEs, some of which can be severe [16–19, 63, 64].

YNBY is known in the fields of hemostasis and blood circulation to have remarkable curative effects and is widely used in China as an adjuvant therapy with hemoptysis because of its low price and low AE incidence [26]. It consisted of the following herbs: *Panax notoginseng, Radix Aconiti Kusnezoffii, Borneolum Synthcticum, Forest Musk,* and *Rhizoma Paridis* (*Chonglou*). It is reported that YNBY ameliorated inflammation via regulating arachidonic acid metabolism [32], and it could be an efficacious agent for bleeding in adolescents with advanced cancer [65]. Some studies showed that the compounds of YNBY significantly shortened the clotting time in PT testing [66]. *Panax notoginseng*, as the main component of YNBY, was involved in the activation of immune cells through the JAK-STAT pathway, which could promote hematopoiesis [67]. And the active ingredients of *Panax notoginseng* could regulate procoagulant platelet formation, which contributes to hemostasis and thrombosis [37]. YNBY likely exerts hemostatic effects by activating platelet aggregation and increasing platelet numbers. Despite its wide use, YNBY treatments in China have not been systematically studied and reviewed. The results of this study provided scientific and rigorous evidence-based medical evidence for the efficacy and safety of Yunnan Baiyao in the treatment of hemoptysis.

### 5.1. Strengths and Limitations

The strengths of this study are as follows: (1) this is the first systematic review of YNBY treatments for hemoptysis. A wide range of search terms were used, and a comprehensive systematic search of various databases was conducted. Currently, there are no guidelines that provide treatment recommendations and strategies for hemoptysis. Current treatment recommendations are based on the published literature. This analysis of existing clinical trials is intended to begin filling in gaps in the literature regarding hemoptysis therapy; (2) RCT selection and data quality evaluation confirmed that YNBY can be used as adjuvant therapy for patients with hemoptysis.

The limitations of this study are as follows: (1) there are key sources of potential bias in the RCTs analyzed. All 13 RCTs in this analysis were performed in China. No RCTs of YNBY in the treatment of hemoptysis have been found to have originated in countries outside of China. The methodological quality of the RCTs was generally low. None of the RCTs mention blinding methods or allocation concealment. These RCT characteristics could have led to biases in our analysis, including selective bias, implementation bias, and measurement bias; (2) the RCTs in this analysis have small sample sizes and are single-center studies, which could result in inaccurate outcome values; (3) most of the RCTs assessed included all causes of hemoptysis, including tuberculosis and bronchiectasis. Because of the limited number of RCTs and the small population sizes assessed, treatment efficacy on hemoptysis from specific causes was not determined; (4) unpublished material was not included, which is a potential limitation of this systematic review. We leave these possible improvements to future studies; (5) none of the RCTs mention results from follow-up visits such that the long-term efficacy and safety of YNBY treatment cannot be assessed.

## 6. Conclusion

The results of this study demonstrate that YNBY + CPT treatment significantly improved several outcomes of patients with hemoptysis: reduced hemoptysis volume; shortened the duration of hemoptysis; shortened the duration of hospitalization; and reduced the incidence of AEs. YNBY + CPT treatment provided no significant difference in the number of cases requiring endotracheal intubation. The results of this study may provide a reliable reference for the application of YNBY in clinical practice. However, the methodological quality of the RCTs was generally low. There were no follow-up visits in the included RCTs, and the RCTs in this analysis have small sample sizes and are single-center studies. In order to promote the applicability of the proposed therapy, it is highly essential to carry out further multicenter, large sample-sized clinical trials. Furthermore, double-blind placebo-controlled randomized clinical trials on TCM should be conducted to effectively improve research quality. Last but not least, the safety of TCM needs to be investigated, and the relevant data should be collected and analyzed systematically.

## Figures and Tables

**Table 1:** Characteristics of the included trials.

Reference	Sample size		Intervention		Age: mean ± SD		Course of treatment		Causes of hemoptysis	Dose and frequency of YNBY (oral)	Outcomes of the studies
	E	C	E (YNBY + CPT)	C (CPT)	E	C	E	C			
Huang [47]	50	50	YNBY + pituitrin or YNBY + phentolamine	Pituitrin or phentolamine	43.4 ± 4.6	2 w	2 w	Bronchiectasis	0.5 g, qid (powder, taken with warm water)	Hemoptysis volume, duration of hemoptysis, number of cases requiring endotracheal intubation	
Lin [48]	42	40	YNBY + pituitrin	Pituitrin	47.7	44.9	1 w	1 w	Tuberculosis	1.5 g, bid (capsule, swallowed)	Effective rate
Liu [22]	45	45	YNBY + pituitrin	Pituitrin	45.37 ± 6.54	45.96 ± 6.15	1 w	1 w	Bronchiectasis	1 g, tid (capsule, swallowed)	Effective rate and adverse events
Peng [49]	34	28	YNBY + pituitrin	Pituitrin	46 ± 13	44 ± 13	1 w	1 w	Bronchiectasis	1 g, tid (capsule, swallowed)	Effective rate, duration of hemoptysis, and adverse events
Qu [40]	50	50	YNBY + carbazochrome sodium sulfonate	Carbazochrome sodium sulfonate	50.23 ± 5.67	49.67 ± 5.59	2 w	2 w	Any causes	0.5 g, tid (capsule, swallowed)	Effective rate, hemoptysis volume, duration of hemoptysis, number of cases requiring endotracheal intubation, and adverse events
Rong [50]	126	126	YNBY + phentolamine	Phentolamine	51.23 ± 3.25	51.30 ± 3.22	5 d	5 d	Bronchiectasis	0.5 g, tid (capsule, swallowed)	Effective rate, hemoptysis volume, duration of hemoptysis, duration of hospitalization, and adverse events
Ruan [51]	125	98	YNBY + pituitrin	Pituitrin	NA	NA	1 w	1 w	Tuberculosis	0.5 g, tid (capsule, swallowed)	Effective rate
Su [52]	68	60	YNBY + pituitrin	Pituitrin	38		2 w	2 w	Tuberculosis	0.5 g, tid (capsule, swallowed)	Effective rate
Wang [53]	23	23	YNBY + phentolamine	Phentolamine	51.2		1 w	1 w	Bronchiectasis	0.5 g, tid (capsule, swallowed)	Effective rate, hemoptysis volume, duration of hemoptysis, and duration of hospitalization
Yan [41]	40	32	YNBY + phentolamine	Phentolamine	NA	NA	5 d	5 d	Any causes	0.5 g, tid (capsule, swallowed)	Effective rate and adverse events
Yang [55]	45	45	YNBY + phentolamine	Phentolamine	50.9 ± 7.4	50.1 ± 6.6	1 w	1 w	Bronchiectasis	0.5 g, tid (capsule, swallowed)	Effective rate
Xi [54]	33	33	YNBY + phentolamine	Phentolamine	56.7 ± 5.2	55.3 ± 4.9	3 d	3 d	Bronchiectasis	0.5 g, tid (capsule, swallowed)	Effective rate and adverse events
Zhou [56]	36	32	YNBY + pituitrin	Pituitrin	38.5		2 w	2 w	Tuberculosis	0.5 g, tid (capsule, swallowed)	Effective rate, hemoptysis volume, and duration of hemoptysis

C: control group; E: experiment group; d: days; w: weeks; YBNY: Yunnan Baiyao; CPT: conventional pharmaceutical treatment; SD: standard deviation, bid: twice a day; tid: three times a day; qid: four times a day; NA: not applicable.

## Supplementary Materials

Supplementary Materials Supplementary File 1: the PICO framework. Supplementary File 2: the search strategy.
