# Supplementary material for: Yunnan Baiyao Adjuvant Treatment for Patients with Hemoptysis: A Systematic Review and Meta-Analysis
Source: Evid Based Complement Alternat Med. 2022 Feb 22;2022:4931284. doi: 10.1155/2022/4931284 (PMC8888054; doi:10.1155/2022/4931284)
Supplement: Supplementary Materials — Supplementary File 1: the PICO framework. Supplementary File 2: the search strategy. [file 4931284.f1.zip › 4931284.f1/PICO framework.docx]

| Population | Hemoptysis OR Hemoptyses OR Hemoptoe OR Spit blood OR Haemoptysis OR Coughing up blood OR Emptysis |
| --- | --- |
| Intervention | Yunnanbaiyao OR Yunnan Baiyao OR Yun nan bai yao OR YNBY OR Baiyao OR Yunnanbaiyao capsule OR Yunnanbaiyao capsules |
| Comparison | Conventional pharmaceutical treatment OR Pituitrin OR Phentolamine OR Carbazochrome sodium sulfonate |
| Outcome | Effective rate OR Hemoptysis volume OR Volume of hemoptysis OR Amount of hemoptysis OR Duration of hemoptysis OR Duration of hospitalization OR Length of stay OR Hospital stay OR Length of hospital stay OR Hospitalization OR Hospital stay duration OR Number of cases requiring endotracheal intubation OR Need for endotracheal intubation OR Need for tracheal intubation |
