# Supplementary material for: Yunnan Baiyao Adjuvant Treatment for Patients with Hemoptysis: A Systematic Review and Meta-Analysis
Source: Evid Based Complement Alternat Med. 2022 Feb 22;2022:4931284. doi: 10.1155/2022/4931284 (PMC8888054; doi:10.1155/2022/4931284)
Supplement: Supplementary Materials — Supplementary File 1: the PICO framework. Supplementary File 2: the search strategy. [file 4931284.f1.zip › 4931284.f1/Supplementary file 1 search strategy.docx]

**Pubmed**

((((((((Hemoptysis[Title/Abstract]) OR (hemoptyses[Title/Abstract])) OR (hemoptoe[Title/Abstract])) OR (spit blood[Title/Abstract])) OR (haemoptysis[Title/Abstract])) OR (coughing up blood[Title/Abstract])) OR (emptysis[Title/Abstract])) AND (((((((yunnanbaiyao[Title/Abstract]) OR (Yunnan Baiyao[Title/Abstract])) OR (Yun nan bai yao[Title/Abstract])) OR (ynby[Title/Abstract])) OR (Baiyao[Title/Abstract])) OR (Yunnanbaiyao capsule[Title/Abstract])) OR (Yunnanbaiyao capsules[Title/Abstract]))) AND ((((((((trial[Title/Abstract]) OR (clinical trials[Title/Abstract])) OR (clinical trial[Title/Abstract])) OR (random[Title/Abstract])) OR (random allocation[Title/Abstract])) OR (therapeutic use[Title/Abstract])) OR (randomized controlled trial[Title/Abstract])) OR (RCT[Title/Abstract]))

**Embase**

#28 #8 AND #16 AND #27

#27 #17 OR #18 OR #19 OR #20 OR #21 OR #22 OR #23 OR #24 OR #25 OR #26

#26 'RCT':ab,ti

#25 'randomized controlled trial':ab,ti

#24 'therapeutic use':ab,ti

#21 'random allocation':ab,ti

#20 'random':ab,ti

#19 'clinical trial':ab,ti

#18 'clinical trials':ab,ti

#17 'trial':ab,ti

#16 #9 OR #10 OR #11 OR #12 OR #13 OR #14 OR #15

#15 'yunnanbaiyao capsules':ab,ti

#14 'yunnanbaiyao capsule':ab,ti

#13 'baiyao':ab,ti

#12 'ynby':ab,ti

#11 'yun nan bai yao':ab,ti

#10 'yunnan baiyao':ab,ti

#9 'yunnanbaiyao':ab,ti

#8 #1 OR #2 OR #3 OR #4 OR #5 OR #6 OR #7

#7 'emptysis':ab,ti

#6 'coughing up blood':ab,ti

#5 'haemoptysis':ab,ti

#4 'spit blood':ab,ti

#3 'hemoptoe':ab,ti

#2 'hemoptyses':ab,ti

#1 'hemoptysis'/exp

**Cochrane library**

#1 hemoptysis 777

#2 hemoptyses 0

#3 hemoptoe 0

#4 spit blood 302

#5 haemoptysis 777

#6 coughing up blood 907

#7 emptysis 3

#8 #1 OR #2 OR #3 OR #4 OR #5 OR #6 OR #7 1928

#9 yunnanbaiyao 1

#10 yunnan baiyao 46

#11 yun nan bai yao 3

#12 ynby 1

#13 baiyao 46

#14 yunnanbaiyao capsule 0

#15 yunnanbaiyao capsules 0

#16 #9 OR #10 OR #11 OR #12 OR #13 OR #14 OR #15 49

#17 #8 AND #16 2

**Web of science**

1 (((((((TS=(hemoptysis)) OR TS=(hemoptyses)) OR TS=(hemoptoe)) OR TS=(spit blood)) OR TS=(haemoptysis)) OR TS=(coughing up blood)) OR TS=(emptysis)

2 (((((((TS=(yunnanbaiyao)) OR TS=(yunnan baiyao)) OR TS=(yun nan bai yao)) OR TS=(ynby)) OR TS=(baiyao)) OR TS=(yunnanbaiyao capsule)) OR TS=(yunnanbaiyao capsules)

3 (((((((TS=(trial)) OR TS=(clinical trials)) OR TS=(clinical trial)) OR TS=(random)) OR TS=(random allocation)) OR TS=(therapeutic use)) OR TS=(randomized controlled trial)) OR TS=(RCT)

4 #1 AND #2 AND #3

**CNKI**

((主题=云南白药) OR (主题=云南白药胶囊)) AND ((咯血) OR (咳血) OR (支气管扩张)) AND ((临床试验) OR (随机对照) OR (随机) OR (临床观察) OR (疗效) OR (试验))

**Wangfang**

((题名或关键词:(云南白药) or 题名或关键词:(云南白药胶囊)) and ((题名或关键词:(咯血) or 题名或关键词:(咳血) or 题名或关键词:(支气管扩张)) and ((题名或关键词:(临床试验) or 题名或关键词:(随机对照) or 题名或关键词:(随机) or 题名或关键词:(临床观察) or 题名或关键词:(疗效) or 题名或关键词:(试验))

**CQVIP**

(M=云南白药 OR 云南白药胶囊) AND (M=咯血 OR 咳血 OR 支气管扩张) AND (M=临床试验 OR 随机对照 OR 随机 OR 临床观察 OR 疗效 OR 试验)

**Sinomed**

4 (#3) AND (#2) AND (#1)

3 "临床试验"[中文标题:智能] OR "随机对照"[中文标题:智能] OR "随机"[中文标题:智能] OR "临床观察"[中文标题:智能] OR "疗效"[中文标题:智能] OR "试验"[中文标题:智能]

2 "咯血"[中文标题:智能] OR "咳血"[中文标题:智能] OR "支气管扩张"[中文标题:智能]

1 "云南白药"[中文标题:智能] OR "云南白药胶囊"[中文标题:智能]
